# Urban–rural structuring of mosquito assemblages in Moyen-Ogooué, Gabon reveals widespread dominance of Aedes albopictus

**DOI:** 10.1038/s41598-026-52848-2

**Published:** 2026-06-16

**Authors:** Gédéon Prince Manouana, Fanny Hellhammer, Ynous Djida, Maminirina Fidélis Ambinintsoa, Moustapha Nzamba Maloum, Terence Stravensky Boussougou-Sambe, Barclaye Ngossanga, Jeannot Fréjus Zinsou, Peter G Kremsner, Julien Zahouli Bi Zahouli, Steffen Borrmann, Stefanie C Becker, Ayola Akim Adegnika

**Affiliations:** 1 Centre de Recherches Médicales de Lambaréné, Lambaréné, Gabon; 2 Institute for Tropical Medicine, University of Tübingen, Tübingen, Germany; 3 Research Center for Emerging Infection and Zoonoses, University of Veterinary Medicine Hanover, Hanover, Germany; 4 Working group for vector associated biodiversity and infection, University of Veterinary Medicine Hanover, Hanover, Germany; 5 Laboratoire de Géomatique, de Recherches Appliquées et de Conseils, Omar Bongo University, Libreville, Gabon; 6 German Center for Infection Research, Tübingen, Germany; 7 Centre Suisse de Recherches Scientifiques en Côte d’Ivoire, Abidjan, Côte d’Ivoire

**Keywords:** Mosquito diversity, Arboviral vectors, Species distribution, Seasonal variation, Urban - rural gradient, Gabon, Diseases, Ecology, Ecology, Zoology

## Abstract

Mosquito-borne arboviruses such as dengue, chikungunya, Zika, yellow fever, West Nile and Rift Valley fever circulate widely in Central Africa, yet vector ecology, especially outside major cities, remains poorly characterized. Between March 2023 and January 2025, we conducted bimonthly entomological surveys to investigate the ecological patterns of mosquito communities across an urban (Lambaréné), peri-urban and rural gradient in Moyen-Ogooué province, central Gabon. Larvae were sampled with WHO ovitraps, and adult mosquitoes were collected using light traps and Prokopack aspirators. In total, 22,216 mosquitoes were collected, representing 21 species from 8 genera. *Aedes* (57.2%) and *Culex.* (23.3%) predominated, alongside *Mansonia.* (16.3%) and lower abundances of *Anopheles*, *Lutzia*, *Coquillettidia*, *Uranotaenia* and *Erethmapodites*. Three species dominated overall: *Ae. albopictus* (52.5%), *Ma. uniformis* (16.2%) and *Cx. quinquefasciatus* (11.3%). Six species have been firstly reported in Gabon including *Ae. argenteopunctatus*, *Ae. opok*, *Cx. bitaeniorhynchus*, *Cx. giganteus*, *Cx. poicilipes*, and *Cx. tritaeniorhynchus*. Rural areas harboured the highest richness (up to 21 species), including numerous rare and functionally specialized taxa. Urban communities were species-poor and strongly dominated by *Ae. albopictus* and *Cx. quinquefasciatus*. Diversity indices and NMDS ordination showed clear, seasonally modulated assemblages across landscapes, with peri-urban sites forming ecological transition zones with intermediate species richness and dominance levels. These findings validate and extend evidence for an *Aedes* and *Culex.* species shift and reveal a highly structured, seasonally dynamic mosquito community in Gabon. Crucially, the coexistence of dominant vectors with a broad array of less common species shows that arboviral transmission potential in central Gabon is shaped by the entire mosquito community. Biodiversity-wide surveillance, not only of major vectors, is therefore essential for understanding and anticipating arbovirus circulation in this ecologically complex region.

**Supplementary Information:**

The online version contains supplementary material available at 10.1038/s41598-026-52848-2.

## Introduction

Across Central Africa, six medically important mosquito-borne arboviruses, chikungunya virus (CHIKV), dengue virus (DENV), West Nile virus (WNV), yellow fever virus (YFV), Zika virus (ZIKV), and Rift Valley fever virus (RVFV) circulate, with CHIKV and DENV detected in all countries of the subregion and all six documented in Gabon^1,2^. Infections caused by these viruses result in substantial morbidity and occasionally severe outcomes in Gabon; however, their true burden remains underestimated due to diagnostic limitations and clinical overlap with other febrile illnesses, particularly malaria^2–4^. Evidence of past outbreaks and ongoing viral circulation highlights a sustained risk of transmission, likely exacerbated by rapid urbanization, population mobility, and favorable environmental conditions for vectors^5–8^. Their transmission is tightly linked to the ecology of mosquito vectors (Culicidae), which connect sylvatic, peri-domestic, and urban cycles and bridge wildlife, livestock, and humans^9^. In this region, rapid land-use change, deforestation, and urban expansion are degrading landscape integrity and reshaping mosquito habitats, thereby altering species composition and abundance across rural, peri-urban, and urban mosaics^10,11^. These shifts in mosquito communities, particularly among competent *Aedes* and *Culex.* vectors, are likely to modulate arbovirus spillover risk and the spatial pattern of human infection^12^, underscoring the need for detailed ecological data on mosquito diversity in Central African settings.

Gabon, located in the Congo Basin with its tropical rainforest, offers an exceptional context for studying these dynamics. This country harbors a wide variety of mosquito species across several genera, including *Aedes*, *Culex.*, *Anopheles*, *Mansonia.* and *Coquillettidia*, each adapted to specific ecological niches^5,13,14^. However, most existing studies have focused on epidemic-related contexts or the capital of Gabon, Libreville, leaving a large part of the country, particularly ecologically diversity in intermediate regions, are under-explored^15,16^. Given that arboviruses circulate in both urban and rural areas of Gabon, mosquito communities across these landscapes are likely to contribute to local transmission dynamics.

The province of Moyen-Ogooué, located in central Gabon, represents an ecological transition zone between dense equatorial rainforest and more open savannah landscapes. It encompasses a wide variety of habitats, including riparian forests, floodplains, agricultural land, peri-urban areas and inland forest ecosystems. This environmental heterogeneity creates a variety of breeding sites for larvae, ranging from tree holes and bamboo stumps to artificial containers and swampy areas, each offering conditions suitable for different mosquito communities^17^. In addition, the presence of large river systems such as the Ogooué River introduces dynamic ecological gradients that can influence mosquito population structure, dispersal, and infection rates^18^.

To date, only one entomological survey has been conducted in this area (November 2019–March 2021) and relying exclusively on electrical aspirators. That study detected only *Aedes (Ae.) albopictus* and *Ae. aegypti*, with *Ae. albopictus* predominant^19^. Although this study offered an initial snapshot of local vector populations, its narrow taxonomic scope left broader mosquito biodiversity unexplored - including species that modulate vector dynamics through larval competition or predation (e.g., *Lutzia* spp.). In addition, the absence of screening for other key arbovirus vectors, such as *Culex (Cx.) quinquefasciatus*, limits our understanding of the region’s entomological landscape and its implications for arbovirus transmission.

Here, we characterize mosquito diversity, abundance, and spatial distribution across the heterogeneous landscapes of Moyen-Ogooué, integrating urban, peri-urban, and rural settings. By linking species composition to landscape features, this study provides essential ecological context for assessing arboviral transmission risk in central Gabon.

## Methods

### Study areas

**Fig. 1:**
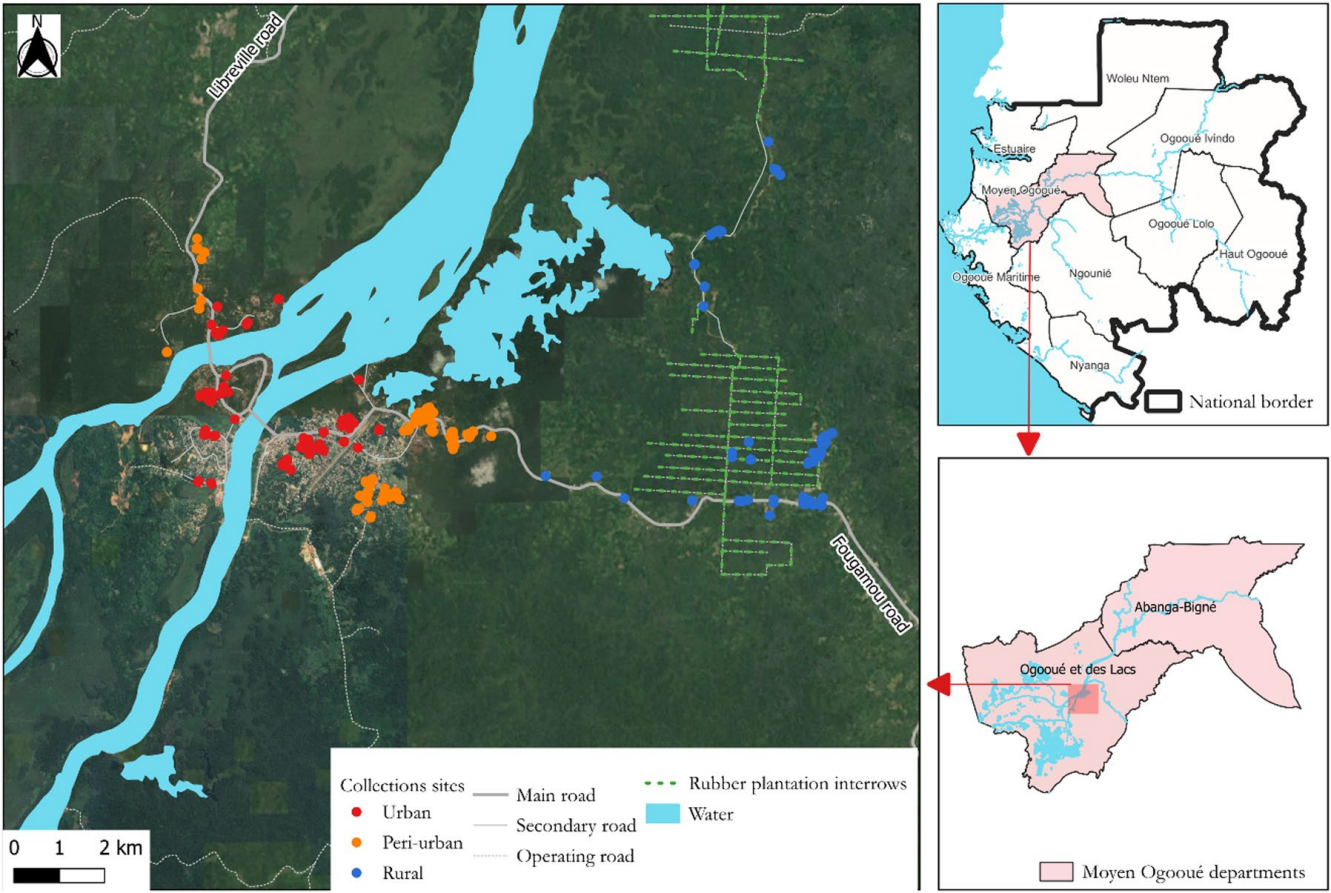
Study area and sampling sites in Moyen-Ogooué province, Gabon. The map depicts Lambaréné and its surrounding landscapes within Moyen-Ogooué province. Sampling locations are marked by colored points representing the three ecological zones: urban (red), peri-urban (orange), and rural (blue). Each site was visited bimonthly between March 2023 and January 2025 for mosquito collection.

The study was conducted in various locations in the province of Moyen-Ogooué, located in central Gabon. The provincial capital, Lambaréné, is located approximately 250 km south of Libreville, on the banks of the Ogooué River. Lambaréné has a population of approximately 40,000 and is bordered by two important agricultural areas: oil-palm plantations approximately 56 km to the north and rubber plantations 25–30 km to the south (Fig. 1).

The study site experiences four distinct seasons each year, characterised by two dry seasons and two rainy seasons. For this study, we defined the rainy seasons as periods when monthly rainfall exceeded 200 mm (October to December and March to June), and the dry seasons as those when monthly rainfall fell below 200 mm (January to February and July to September).

## Study design

Entomological surveys were conducted in Lambaréné (urban) and its surrounding peri-urban areas, and neighboring rural zones between March 2023 and January 2025 every two months, covering both wet and dry seasons. During the study period, sampling was carried out at three defined sites within each study area, namely urban, peri-urban and rural. Every two months, mosquitoes adults, larvae and eggs were collected at each site using the three sampling methods (light traps, electrical aspirators, and ovitraps).

Urban, peri-urban and rural areas were distinguished based on the socio-spatial characteristics of communities and their level of infrastructure provision (Fig. 1). In each zone, three neighborhoods (urban and peri-urban) or three villages (rural) were purposively selected to represent typical socio-environmental conditions. Within each site, four households were chosen for traps placement based on accessibility and willingness to participate, ensuring that the selected households were spaced apart to reduce interference during trap placement.

The urban area corresponds to the city centre and is characterized by high population density and a concentration of public administrative structures (town hall, decentralised government services, police stations), socio-economic infrastructure (referral hospitals, secondary schools, shops, markets) and modern water and electricity supply networks.

The peri-urban area represents the outlying neighborhoods on the edge of the city center, forming a transition zone between urban and rural areas. It is characterized by less dense housing and a partial and uneven presence of basic infrastructure (limited access to drinking water and electricity).

The rural zone encompasses villages situated approximately 15 km from the urban centre on the road Fougamou, characterized by low population density.

## Entomological sampling

Larvae were collected using WHO standardized ovitraps in each study site per survey^20^. Ovitraps were filled out at ¾ volume with tap water and left in the field for a week for each survey. Then, the ovitrap paddles and contents (water, eggs and larvae) were sampled and stored separately in labelled plastic cups. Adult mosquitoes were collected alive using light traps (LTs) (BioQuip Products, Inc., Rancho Dominguez, CA, USA) and Procopack-type aspirators (BioQuip Products, Inc., Rancho Dominguez, CA, USA). The light traps were placed in and next to households for approximately 15 h from 4 p.m. to 7 a.m. per survey. Meanwhile, resting adult mosquitoes were collected next to households using Procopack-type aspirators in the morning from 6 a.m. to 9 a.m. Collected mosquitoes were placed separately into labelled plastic cups covered with netting.

## Laboratory procedures

All mosquito samples were immediately transported in cool boxes to the field laboratory for further processing. Ovitrap paddles were dried for five days under ambient conditions. Then, each paddle was immersed individually in white-bottomed trays filled up to 75% capacity with distilled water for egg hatching. Egg-derived and field-collected larvae and pupae were counted, placed separately in plastic cups filled with water and covered with fine mesh and fed daily with Tetramin Fish Baby Food until adult emergence. Emerged and field-collected adults were identified morphologically to species and sex using available taxonomic keys^21,22^.

### Data analysis

All analyses were performed using R software version 4.0.2 (R Core Team, R Foundation for Statistical Computing, Vienna, Austria), and mapping was performed using QGIS (version 3.44, Long Term Release). Specific abundances were transformed according to Hellinger before multivariate analyses.

To characterize dominance structure and evenness within mosquito assemblages, rank–abundance (Whittaker) curves were generated. Abundances were first aggregated by taxon within each grouping variable (season or locality), and only taxa with non-zero abundances were retained. Taxa were then ranked in descending order of total abundance within each group. Relative abundances were calculated by dividing each taxon’s abundance by the total abundance of the respective group. Rank–abundance curves were plotted using relative abundance against species rank (from most to least abundant) on a linear scale (0–100%). Differences in curve shape were interpreted descriptively: steep slopes indicate strong dominance by a few taxa and low evenness, whereas flatter curves reflect more even species distributions.

Non-metric multidimensional scaling (NMDS) was used to visualise differences in mosquito community composition among localities and seasons. A community matrix was assembled by aggregating abundances per taxon within each locality × season group and reshaping the data to a wide site-by-taxon format, with absent taxa coded as zeros. Bray–Curtis dissimilarities were calculated from the resulting abundance matrix and ordinated in two dimensions (k = 2) using the metaMDS function (vegan package). To ensure a stable solution, the algorithm was run with up to 100 random starts (trymax = 100) and a fixed random seed for reproducibility. Automatic data transformation within metaMDS was disabled (autotransform = FALSE) because transformations were handled prior to ordination. Ordination fit was assessed using the stress value. For visualisation, site scores (NMDS1–NMDS2 coordinates) were extracted and plotted with points coloured by locality and shaped by season. Group dispersion was summarized using dashed confidence ellipses (68% level) drawn for each locality.

Standardised biodiversity indices (species richness, abundance, dominance, evenness) were calculated for each ecological group^23^. These indices include Species richness, defined as the total number of species detected irrespective of their abundance, and the Shannon diversity index (H′), which integrates both species richness and the relative distribution of abundances among species. Univariate analyses were conducted using non-parametric tests (Kruskal–Wallis). Abundance data were explored graphically using box plots and a heat map to visualize variations in the number of individuals according to sampling methods and species. The statistical significance threshold for all tests was set at 0.05.

## Results

### Mosquito composition and relative abundance

A total of 22,216 mosquitoes were collected, encompassing 21 species across 8 genera (overview in Table S1). The identified genera included *Aedes* (6 species), *Culex* (8 species), *Anopheles* (4 species), *Mansonia* (2 species), and one species each from *Lutzia*, *Coquillettidia*, *Uranotaenia*, and *Erethmapodites*. The most abundant genus was *Aedes*, which represented 57.18% (*n* = 12,704) of the total collection. This was followed by *Culex* with 23.25%.

(*n* = 5,166), and *Mansonia*, accounting for 16.29% (*n* = 3,618). *Anopheles* mosquitoes comprised 2.56% (*n* = 568) of the specimens. In contrast, *Uranotaenia*, *Coquillettidia*, *Lutzia*, and *Erethmapodites* were much less represented, contributing only 0.46%, 0.17%, 0.06%, and 0.03% of the total collection, respectively. Three dominant species were identified: *Ae. albopictus* was the most prevalent, making up 52.53% (*n* = 11,671) of the total specimens, followed by *Mansonia* (*Ma.) uniformis* with 16.18% (*n* = 3,594) and *Cx. quinquefasciatus* with 11.29% (*n* = 2,508).

## Distribution of sampled mosquito species across developmental stages

**Fig. 2:**
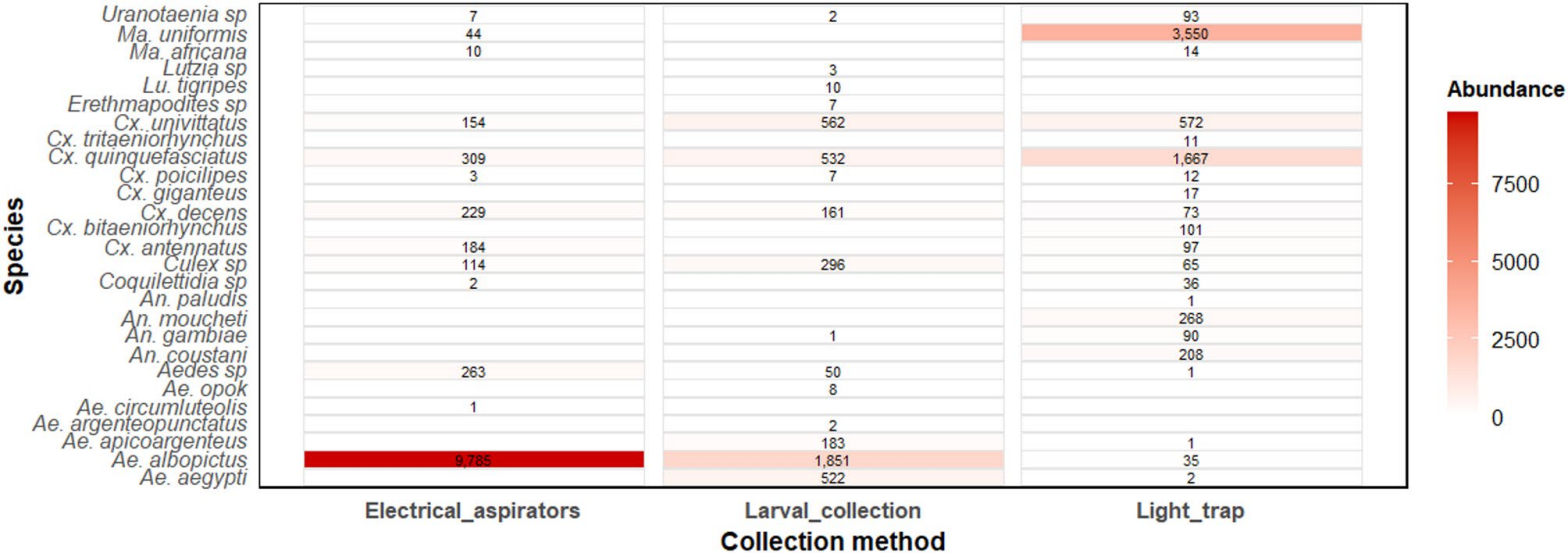
Heat map illustrating variation in mosquito abundance across species and sampling methods. Each cell represents the number of individuals collected for a given species–method combination, with a colour gradient from white (low abundance) to deep red (high abundance).

Capture patterns differed markedly among the three sampling methods (light traps, electrical aspirators, and ovitraps), with clear taxon-specific profiles (Table S2 and Fig. 2). Of the 22,216 mosquitoes collected in this study, 18,019 were captured as adults using two types of traps, light traps (*n* = 6,914) and electrical aspirators (*n* = 11,105), while the remaining 4,197 were larvae and pupae that developed into adults.

Several *Aedes* species were recorded predominantly or exclusively in their immature stages, including *Ae. aegypti* (99.6% of detections from larvae), *Ae. apicoargenteus* (99.5%), *Ae. Argenteopunctatus* (100%), and *Ae. opok* (100%). *Erethmapodites sp.* and *Lutzia* (including *Lutzia tigripes*) were detected exclusively in larval collections and were not recorded among adult captures.

*Ae. albopictus* showed a contrasting pattern, with most individuals captured as adults using electrical aspirators (83.8%), followed by larval sampling (15.9%), and only rarely detected in light traps. Light traps were the primary method for collecting *Anopheles* and *Mansonia* species: *Anopheles coustani*, *Anopheles moucheti*, *Anopheles paludis*, and *Ma. uniformis* were detected almost exclusively with light traps (> 98%), and similar patterns were observed for *Coquillettidia* sp. and *Uranotaenia* sp.

Among *Culex* species, collection profiles varied. *Cx. quinquefasciatus* was captured mainly by light traps (66.5%), followed by larval collections (21.2%), whereas *Cx. antennatus* showed a more even distribution between aspiration (65.5%) and light-trap sampling (34.5%). *Cx. decens*, *Cx. poicilipes*, and *Cx. univittatus* were collected by all three methods, with *Cx. univittatus* showing near-equal representation in light traps (44.4%) and larval samples (43.6%).

### Geographic distribution of mosquito vectors

**Fig. 3:**
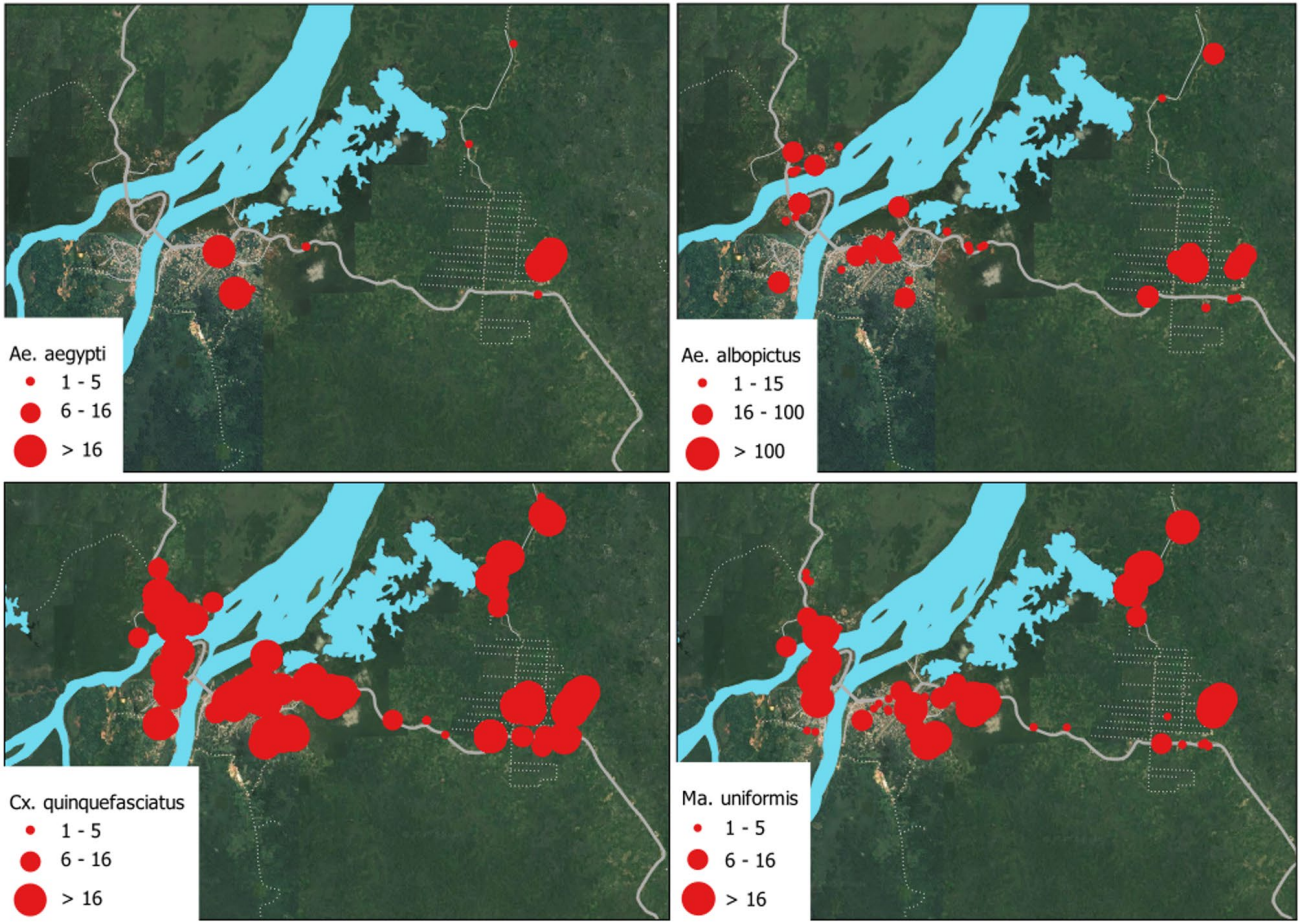
Spatial distribution of the four most predominant mosquito species according to the study areas (Urban to rural areas).

Across the urban–peri-urban–rural gradient, mosquito communities were structured by a small number of highly abundant species against a background of numerous less common taxa (Fig. 3). *Ae. albopictus* and *Cx. quinquefasciatus* were the most prominent contributors to overall abundance. *Ae. albopictus* dominated rural (63.7%) and urban (46.8%) communities but was less frequent in peri-urban areas (6.2%). In contrast, *Cx. quinquefasciatus* was strongly associated with more anthropogenic environments, representing 32.6% of urban and 27.7% of peri-urban collections but only 2.0% of rural specimens. *Ma. uniformis* was another major contributor, especially in peri-urban areas, where it comprised 40.3% of all mosquitoes, and remained important in rural (13.1%) and, to a lesser extent, urban (9.3%) habitats. Several species showed broad ecological distribution with moderate abundances: *Ae. aegypti* occurred at low but similar proportions across all three landscapes (1.4–2.7%). *Cx. decens* was recorded mainly in urban and rural areas.

*Cx. univittatus* was detected in all habitats but was more frequent in peri-urban (10.1%) and rural (6.0%) sites. Rural areas harboured the highest number of species with 24 taxa, whereas urban and peri-urban landscapes each hosted 19 species, underscoring greater taxonomic richness in the more natural parts of the landscape.

**Fig. 4:**
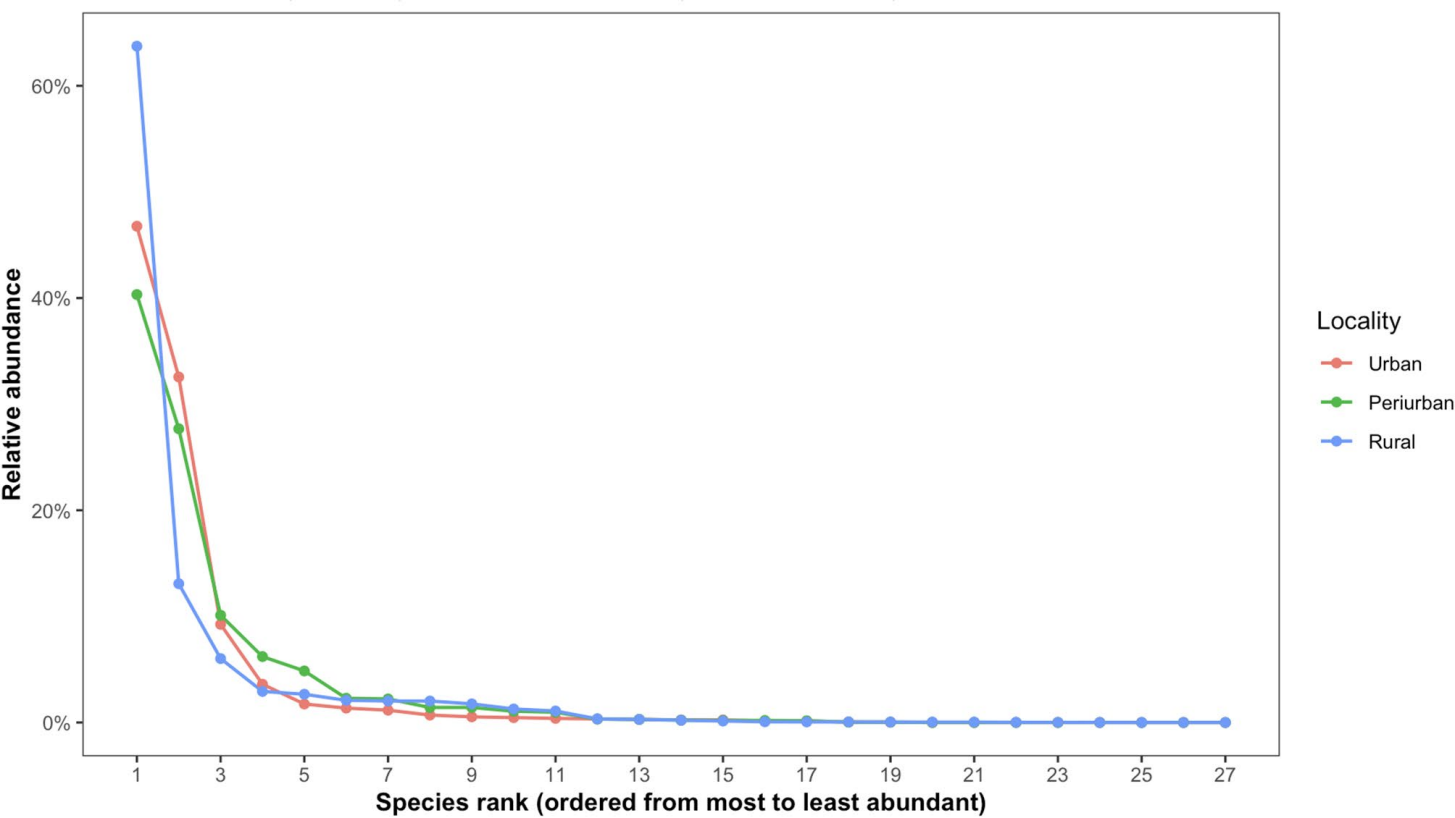
Rank–abundance (Whittaker) curves by habitats. Species are ordered along the x-axis from the most to the least abundant within each locality. The y-axis shows relative abundance expressed as percentages. The slope of each curve reflects community evenness: steeper curves indicate strong dominance by a few species, whereas flatter curves indicate a more even distribution of abundances among species. Differences in curve length represent variation in species richness among localities.

In addition to these dominant and broadly distributed taxa, a suite of species occurred at low frequencies in this study. These included several *Aedes* species detected almost exclusively in rural environments, *Ae. argenteopunctatus*, *Ae. circumluteolis*, and *Ae. opok*, as well as *Anopheles paludis*, *Coquillettidia* spp., *Cx. giganteus*, *Cx. poicilipes*, *Cx. tritaeniorhynchus*, *Erethmapodites* sp., and the predatory taxa *Lutzia* sp. and *Lutzia tigripes*, all of which together represented only a small fraction of the total collection. *Ma. africana* and *Uranotaenia* spp. were likewise infrequent, though the latter occurred at low but comparable proportions across all three landscapes. Overall, the data indicate that urban communities were dominated by a few well-adapted vector species, peri-urban areas are characterized by co-dominance of *Culex* and *Mansonia*, and rural sites combine extremely high *Ae. albopictus* abundance with the richest assemblage of less common and habitat-specialist species (Fig. 4 and Table S1).

### Seasonal variation in mosquito relative abundance and species diversity

**Fig. 5:**
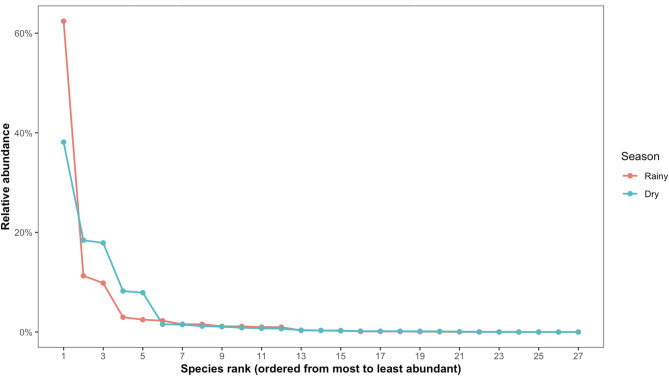
Rank–abundance (Whittaker) curves by seasons. Species are ranked along the x-axis from the most to the least abundant within each season. The y-axis displays relative abundance expressed as percentages. The slope of each curve reflects community evenness: steeper slopes indicate dominance by a few species, whereas flatter slopes indicate a more even distribution of species abundances. Differences in the length of the curves represent variation in species richness between the rainy and dry seasons.

Across seasons, mosquito communities were shaped by a small number of dominant species whose dynamics shifted markedly with rainfall. Overall mosquito abundance increased significantly during the rainy season (*p* = 0.0021), a trend also evident in both urban (*p* = 0.0085) and rural (*p* = 0.0034) sites. During the rainy period, *Ae. albopictus* was overwhelmingly dominant, accounting for 62.4% of all individuals, far exceeding the contributions of *Ma. uniformis* (11.3%) and *Cx. quinquefasciatus* (9.8%), the next most abundant taxa. In contrast, the dry season showed a markedly different structure: *Ma. uniformis* emerged as the dominant species (38.1% of all dry season captures), followed by *Cx. univittatus* (18.4%) and *Cx. quinquefasciatus* (17.9%) (Fig. 5 and Table S1). *Ae. albopictus* declined to 8.2% of dry-season collections, while *Ae. aegypti* increased proportionally from 1.1% (rainy) to 7.9% (dry), despite remaining a minor overall contributor. Several species, such as *Anopheles gambiae s.l.*, *Anopheles moucheti*, *Cx. bitaeniorhynchus*, *Coquillettidia* sp., and *Uranotaenia* sp., were present in both seasons with moderate and relatively stable frequencies, each contributing between roughly 0.3–1.5% per season. Overall, rainy-season communities were defined by the explosive dominance of rainfall-responsive species, especially *Ae. albopictus*, whereas dry-season assemblages were more evenly structured, with *Ma. uniformis*, *Cx. univittatus*, and *Cx. quinquefasciatus* forming the core of the community.

Many species were detected only at very low frequencies, allowing only cautious statements about their seasonal occurrence. Several taxa appeared exclusively or predominantly during the rainy season, including *Ae. argenteopunctatus*, *Ae. circumluteolis*, *Cx. antennatus*, and *Cx. tritaeniorhynchus*, all of which were recorded only in small numbers. Conversely, a few rare taxa were found only during the dry season, such as *Anopheles paludis*, *Erethmapodites* sp., *Lutzia* sp., and *Lutzia tigripes*. Other infrequent species, *Ae. opok*, *Cx. giganteus*, and *Cx. poicilipes*, were observed in both seasons but remained numerically scarce throughout. All of these taxa were represented by small numbers of individuals and occurred sporadically across seasons and habitats.

### Seasonal variation across landscapes types

Patterns of richness and diversity varied strongly across landscapes and seasons). Species richness was consistently highest in rural sites (21 species in the rainy season and 19 in the dry season). In the rainy season, richness was slightly higher in urban areas (19 species) than in peri-urban areas (18 species), whereas in the dry season, peri-urban areas (15 species) had higher richness than urban environments (14 species), where the lowest values were recorded. Total mosquito abundance was highest in rural areas during the rainy season (12,992 individuals), compared with 3,343 in urban and 1,828 in peri-urban sites.

The Shannon diversity index (H′) revealed additional differences in community structure. Urban areas showed slightly higher Shannon index values in the dry season.

(H′ = 1.44) than in the rainy season (H′ = 1.34), indicating reduced dominance outside the peak rainfall period. Peri-urban sites exhibited the highest Shannon index overall, particularly during the rainy season (H′ = 1.87), reflecting the contribution of several co-occurring taxa to overall community structure, while still maintaining a moderate diversity in the dry season (H′ = 1.39). In contrast, rural sites showed the lowest Shannon index during the rainy season (H′ = 1.19) despite having the highest species richness, indicating strong dominance by a single species. During the dry season, the Shannon index in rural areas increased substantially (H′ = 1.73), reflecting a more even distribution of individuals across taxa.

**Fig. 6:**
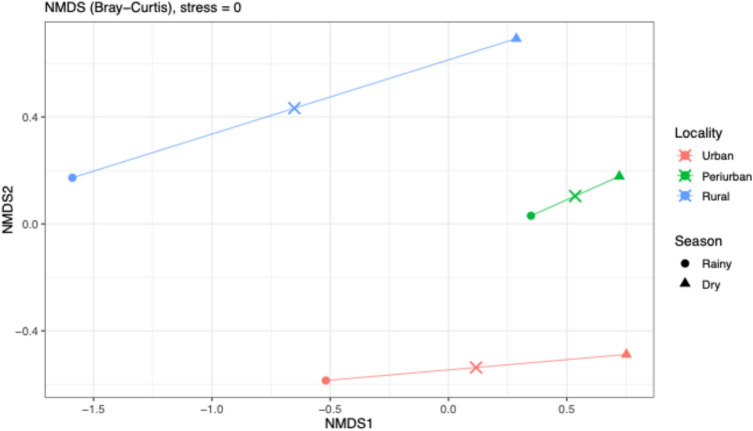
Non-metric multidimensional scaling (NMDS) ordination based on Bray–Curtis dissimilarities of mosquito assemblages across localities (Urban, Peri-urban, Rural) and seasons (Rainy, Dry). Spider lines connect seasonal samples within each locality to their centroids (cross symbols). NMDS1 and NMDS2 represent the two ordination axes that summarize multivariate differences in community composition; the relative distances between points reflect dissimilarities in species assemblages (closer points indicate more similar communities). The ordination stress was zero, indicating a perfect two-dimensional representation of sample dissimilarities.

The non-metric multidimensional scaling (NMDS) ordination further supported these seasonal and spatial differences (Fig. 6). In rural areas, the distance between rainy- and dry-season samples was large, indicating strong seasonal turnover in species composition. Peri-urban sites displayed only minor seasonal shifts, while urban sites showed intermediate differences between seasons. Overall, samples from the three landscapes formed distinct clusters in ordination space, with peri-urban assemblages positioned between the urban and rural groups. The near-zero stress value (stress = 0) indicates a clear representation of community dissimilarities in the two-dimensional ordination.

## Discussion

This study provides the first comprehensive characterization of mosquito diversity and spatial structuring across urban, peri-urban, and rural landscapes in the Moyen-Ogooué province of Gabon. By combining larval and adult sampling over two years, we reveal a far richer and more heterogeneous mosquito fauna than previously documented, including clear landscape-specific species assemblages, strong seasonal shifts, and distinct abundance gradients among key arbovirus vectors. These findings fill a critical knowledge gap in a region where arboviruses circulate in both sylvatic and urban cycles, yet detailed ecological data on vector communities outside major cities have been largely lacking. The pronounced differences in diversity, dominance, and community composition across the urban–rural gradient highlight how landscape structure and seasonality shape vector populations, providing important ecological context for understanding arboviral transmission dynamics in Central Africa.

In total, 22,216 mosquitoes were captured, comprising 21 species from across 8 different genera, indicating considerable species richness within the region. The species identified in this study show similarities with what was reported in other regions of Gabon outside Moyen-Ogooué province^1,13,14^. In line with previous studies, *Aedes* and *Culex* were the most abundant genera, including major arbovirus vectors such as *Ae. aegypti*, *Ae. albopictus* or *Cx. quinquefasciatus*^14^. These findings highlight the widespread distribution and epidemiological relevance of these vector species across the country. Although the overall species composition closely resembled patterns reported from other regions of Gabon, our survey also broadened the country’s documented mosquito fauna by detecting several species not previously reported. These included *Ae. argenteopunctatus*, *Ae. opok*, *Cx. bitaeniorhynchus*, *Cx. giganteus*, *Cx. poicilipes*, and *Cx. tritaeniorhynchus*, highlighting the added value of sampling across ecologically diverse habitats. These findings likely reflect previous under-detection rather than true absence and point to a more complex local mosquito community than previously recognized, with ecological variability that may influence arbovirus transmission dynamics. Several of these species, *Cx. tritaeniorhynchus*, *Cx. poicilipes*, and *Cx. giganteus*, although considered secondary vectors, may exert important ecological effects. Their contribution is particularly evident at the larval stage, where competition for space and resources can alter the population trajectories of primary vectors such as *Ae. aegypti* and *Ae. albopictus*^24^. In addition, larvae of the genus *Lutzia* and *Eretmapodites*, known as natural predators of other mosquito larvae, were also encountered. Their presence reflects biological pressure within aquatic habitats and may further modulate vector population dynamics by reducing the survival of co-occurring species^25^. The coexistence of sylvatic *Aedes* species such as *Ae. argenteopunctatus* and *Ae. opok*, together with ecologically plastic *Culex* species including *Cx. bitaeniorhynchus*, *Cx. giganteus*, *Cx. poicilipes*, and *Cx. tritaeniorhynchus*, creates a plausible ecological continuum linking enzootic maintenance in wildlife to epizootic amplification and eventual human and livestock exposure^26–28^. Overall, although the predominance of the *Aedes* and *Culex genera* is consistent with known regional patterns, the occurrence of other genera points to a complex vector ecosystem that requires further ecological and epidemiological research.

The marked suppression of *Ae. aegypti* observed in this study, despite its continued reproduction in the area, stands in sharp contrast to findings from other West and Central African regions with comparable climate, such as Côte d’Ivoire. In these regions, similar trapping approaches typically yield *Ae. aegypti* as the dominant container-breeding species and *Ae. albopictus* remains comparatively rare^29^. The near-complete adult displacement documented here therefore represents not a methodological artifact, but a genuine ecological restructuring of *Aedes* populations within the Moyen-Ogooué region. Our findings provide strong confirmation of the *Aedes* species shift previously reported in Lambaréné, where *Ae. albopictus* had already been identified as the emerging dominant species. By integrating larval, adult and oviposition-based sampling across multiple habitats, our study demonstrates that this shift is not confined to the urban core but extends across peri-urban and rural landscapes, indicating a broad-scale and possibly consolidated replacement event. The detection of *Ae. aegypti* primarily in its immature stages suggests that although the species is still reproducing locally, it fails to establish sizeable adult populations, likely due to competitive suppression by *Ae. albopictus*, a process that has been described in other tropical settings following albopictus invasion. Importantly, the presence of abundant *Ae. albopictus* in natural and semi-natural rural environments further underscores the extent of this ecological transition^30^. It suggests that the ecological context of the Congo Basin, characterized by high humidity, dense vegetation, and complex larval habitats, may offer particularly favorable conditions for *Ae. albopictus*, facilitating its spread into ecological niches traditionally occupied by *Ae. aegypti* (23).

In addition to the pronounced dominance of *Ae. albopictus* over *Ae. aegypti* observed in this study, our results also reveal significant variation in species composition and abundance across landscapes. As expected, species richness was higher in rural and peri-urban areas than in urban areas. This trend is consistent with observations made in other tropical regions, where rural settings generally have more diverse and stable breeding habitats, such as natural water bodies and vegetation cover, which promote greater species diversity^31,32^. Species dominance varied markedly across landscapes, indicating clear ecological structuring along the urban–rural gradient. Urban assemblages were depauperate and dominated by synanthropic vectors, especially *Cx. quinquefasciatus* and *Ae. albopictus*, both well adapted to artificial containers and densely populated environments^33^. This could be the cause of an increased risk of arbovirus transmission, given their preference for human hosts and their high vector competence^34–36^.

Beyond the dominant vectors, landscape differences were further reflected in the distribution of rare and functionally specialized taxa. Rural areas harboured several low-abundance species, including *Ae. opok*, *Ae. argenteopunctatus*, *Cx. giganteus*, and *Erethmapodites* sp., which are typically associated with natural or semi-natural larval habitats and are rarely observed in highly modified urban environments^37^. Larval predators such as *Lutzia* spp. were detected exclusively in rural settings, indicating the presence of more complex trophic interactions in less disturbed habitats. Their absence from urban areas suggests ecological simplification and functional homogenisation of mosquito communities in the city, where assemblages were largely dominated by *Ae. albopictus* and *Cx. quinquefasciatus*.

Peri-urban areas exhibited an intermediate structure, combining urban-adapted vectors with species more commonly associated with rural or semi-natural habitats. This compositional overlap highlights the role of peri-urban landscapes as ecological transition zones where taxa from distinct environmental settings co-occur.

Seasonal fluctuations in mosquito communities are a defining feature of equatorial ecosystems, and the patterns documented in this study conform closely to what is known from comparable tropical regions. As expected, mosquito abundance increased markedly during the rainy season across all landscape types, reflecting the expansion of aquatic larval habitats and improved adult survival under humid conditions^38^. Likewise, genera known to dominate humid tropical environments, *Aedes*, *Culex*, and *Mansonia*, were consistently the most abundant, mirroring patterns described in other parts of Gabon and throughout Central Africa^1,14^. The observed seasonal fluctuations in diversity and evenness, including reduced evenness in rural areas during the rainy season, also align with studies from similar ecological contexts, where a subset of highly opportunistic species proliferates rapidly under favorable hydroclimatic conditions^39^. These findings collectively validate the ecological expectations for mosquito communities in equatorial rainforest zones and demonstrate broad concordance with established literature from the Congo Basin and neighboring regions.

While the overall seasonal patterns align with ecological expectations, several features of the spatial dynamics observed in this study depart from patterns previously described in Gabon and elsewhere in Central Africa^40^. Most notably, *Ae. albopictus* exhibited an unexpectedly strong dominance in rural environments during the rainy season, indicating that this invasive vector has successfully expanded into natural or semi-natural habitats where it was historically scarce^41^. This shift suggests a restructuring of rural mosquito communities, with potential implications for the spatial range and seasonal timing of arboviral transmission. In contrast, *Cx. quinquefasciatus* remained tightly associated with urban and peri-urban settings, reinforcing the strong coupling between anthropogenic landscapes and the ecology of this key vector. Peri-urban areas, where urban and rural taxa consistently co-occurred, emerged as ecologically hybrid zones that may facilitate contact between vectors associated with distinct transmission cycles. Altogether, these patterns demonstrate that the interaction between rainfall and landscape type produces highly structured and non-uniform shifts in community composition, driven by the differential seasonal amplification of dominant vector species across ecological gradients.

This study has several limitations. Bimonthly sampling may have missed short-term fluctuations in mosquito populations, and the three sites per landscape may not fully represent the province. The absence of true replicates in our sampling design limited our ability to perform robust statistical analyses to formally test differences in species abundance across trap types and environmental gradients. While ovitraps, light traps, and Prokopack aspirators captured most species, cryptic or exophilic mosquitoes could be underrepresented. Morphological identification may have introduced observer bias, and molecular approaches were not employed to confirm species identifications. Despite these constraints, the study provides a robust baseline of mosquito community structure and seasonal dynamics across urban–rural gradients.

## Conclusion

Taken together, this study reveals that mosquito communities in Moyen-Ogooué are far more diverse, spatially structured, and seasonally dynamic than previously recognized. By uncovering distinct vector assemblages across the urban–rural gradient and showing how rainfall differentially amplifies key species, our findings demonstrate that arboviral transmission risk in central Gabon is shaped by a combination of landscape heterogeneity and pronounced seasonal forcing. The unexpected rural dominance of *Ae. albopictus* and the clear ecological segregation of *Cx. quinquefasciatus* underscore that vector species are exploiting specific environmental niches, creating shifting windows of vulnerability for human populations. These results highlight the need for surveillance systems that extend beyond major cities and incorporate both seasonal and ecological context, as well as the importance of integrated vector monitoring strategies capable of capturing the full complexity of local mosquito ecosystems. Ultimately, a deeper understanding of these ecological dynamics is essential for anticipating how arboviruses may circulate, expand, or re-emerge in a rapidly changing Central African landscape.

## Electronic Supplementary Material

Below is the link to the electronic supplementary material.

Supplementary Material 1

Supplementary Material 2

## Data Availability

The datasets used and/or analysed during the current study are available from the corresponding author on reasonable request.
