# Supplementary material for: Urban–rural structuring of mosquito assemblages in Moyen-Ogooué, Gabon reveals widespread dominance of Aedes albopictus
Source: Sci Rep. 2026 Jun 16;16:18699. doi: 10.1038/s41598-026-52848-2 (PMC13273180; doi:10.1038/s41598-026-52848-2)
Supplement: Supplementary file 1 — Supplementary Material 1 [file 41598_2026_52848_MOESM1_ESM.docx]

**Table S1. Species, number, and abundance (%) of mosquito vector in the study area (N=22216).**

| **Genus** | **Species** | **Number** | **Genus (%)** | **Species (%)** |
| --- | --- | --- | --- | --- |
|  |  |  |  |  |
| *Aedes* | *aegypti* | 524 | 57.18 | 2.36 |
| *Aedes* | *albopictus* | 11671 |  | 52.53 |
| *Aedes* | *apicoargenteus* | 184 |  | 0.83 |
| *Aedes* | *argenteopunctatus* | 2 |  | 0.01 |
| *Aedes* | *circumluteolis* | 1 |  | 0 |
| *Aedes* | *opok* | 8 |  | 0.04 |
| *Aedes* | *sp* | 314 |  | 1.41 |
| *Anopheles* | *coustani* | 208 | 2.56 | 0.94 |
| *Anopheles* | *gambiae sl* | 91 |  | 0.41 |
| *Anopheles* | *moucheti* | 268 |  | 1.21 |
| *Anopheles* | *paludis* | 1 |  | 0 |
| *Coquilettidia* | *sp* | 38 | 0.17 | 0.17 |
| *Culex* | *antennatus* | 281 | 23.25 | 1.26 |
| *Culex* | *bitaenyrhincus* | 101 |  | 0.45 |
| *Culex* | *decens* | 463 |  | 2.08 |
| *Culex* | *giganteus* | 17 |  | 0.08 |
| *Culex* | *poïcilipes* | 22 |  | 0.1 |
| *Culex* | *quinquefasciatus* | 2508 |  | 11.29 |
| *Culex* | *tritaeniorhynchus* | 11 |  | 0.05 |
| *Culex* | *univittatus* | 1288 |  | 5.8 |
| *Culex* | *sp* | 475 |  | 2.14 |
| *Erethmapodites* | *sp* | 7 | 0.03 | 0.03 |
| *lutzia* | *sp* | 10 | 0.06 | 0.05 |
| *lutzia* | *tigripes* | 3 |  | 0.01 |
| *Mansonia* | *africana* | 24 | 16.29 | 0.11 |
| *Mansonia* | *uniformis* | 3594 |  | 16.18 |
| *Uranotaenia* | *sp* | 102 | 0.46 | 0.46 |
