# Supplementary material for: Urban–rural structuring of mosquito assemblages in Moyen-Ogooué, Gabon reveals widespread dominance of Aedes albopictus
Source: Sci Rep. 2026 Jun 16;16:18699. doi: 10.1038/s41598-026-52848-2 (PMC13273180; doi:10.1038/s41598-026-52848-2)
Supplement: Supplementary file 2 — Supplementary Material 2 [file 41598_2026_52848_MOESM2_ESM.docx]

| **Genus** | **Species** | **Collection methods** | | | | | |
| --- | --- | --- | --- | --- | --- | --- | --- |
|  |  | Light trap  (N=6914) | | Electrical aspirators (N=11105) | | Larval collection (N=4197) | |
|  |  | **n** | **%** | **n** | **%** | **n** | **%** |
| *Aedes* | *aegypti* | 2 | 0.38 | 0 | 0.00 | 522 | 99.62 |
| *Aedes* | *albopictus* | 35 | 0.30 | 9785 | 83.84 | 1851 | 15.86 |
| *Aedes* | *apicoargenteus* | 1 | 0.54 | 0 | 0.00 | 183 | 99.46 |
| *Aedes* | *argenteopunctatus* | 0 | 0.00 | 0 | 0.00 | 2 | 100.00 |
| *Aedes* | *circumluteolis* | 0 | 0.00 | 1 | 100.00 | 0 | 0.00 |
| *Aedes* | *opok* | 0 | 0.00 | 0 | 0.00 | 8 | 100.00 |
| *Aedes* | *sp* | 1 | 0.32 | 263 | 83.76 | 50 | 15.92 |
| *Anopheles* | *coustani* | 208 | 100.00 | 0 | 0.00 | 0 | 0.00 |
| *Anopheles* | *gambiae sl* | 90 | 98.90 | 0 | 0.00 | 1 | 1.10 |
| *Anopheles* | *moucheti* | 268 | 100.00 | 0 | 0.00 | 0 | 0.00 |
| *Anopheles* | *paludis* | 1 | 100.00 | 0 | 0.00 | 0 | 0.00 |
| *Coquilettidia* | *sp* | 36 | 94.74 | 2 | 5.26 | 0 | 0.00 |
| *Culex* | *antennatus* | 97 | 34.52 | 184 | 65.48 | 0 | 0.00 |
| *Culex* | *bitaenyrhincus* | 101 | 100.00 | 0 | 0.00 | 0 | 0.00 |
| *Culex* | *decens* | 73 | 15.77 | 229 | 49.46 | 161 | 34.77 |
| *Culex* | *giganteus* | 17 | 100.00 | 0 | 0.00 | 0 | 0.00 |
| *Culex* | *poïcilipes* | 12 | 54.55 | 3 | 13.64 | 7 | 31.82 |
| *Culex* | *quinquefasciatus* | 1667 | 66.47 | 309 | 12.32 | 532 | 21.21 |
| *Culex* | *tritaeniorhynchus* | 11 | 100.00 | 0 | 0.00 | 0 | 0.00 |
| *Culex* | *univittatus* | 572 | 44.41 | 154 | 11.96 | 562 | 43.63 |
| *Culex* | *sp* | 65 | 13.68 | 114 | 24.00 | 296 | 62.32 |
| *Erethmapodites* | *sp* | 0 | 0.00 | 0 | 0.00 | 7 | 100.00 |
| *lutzia* | *sp* | 0 | 0.00 | 0 | 0.00 | 3 | 100.00 |
| *lutzia* | *tigripes* | 0 | 0.00 | 0 | 0.00 | 10 | 100.00 |
| *Mansonia* | *africana* | 14 | 58.33 | 10 | 41.67 | 0 | 0.00 |
| *Mansonia* | *uniformis* | 3550 | 98.78 | 44 | 1.22 | 0 | 0.00 |
| *Uranotaenia* | *sp* | 93 | 91.18 | 7 | 6.86 | 2 | 1.96 |

**Table S2. Abundance of mosquito species by sampling method**
